# Making base editing accessible: Evaluating computational workflows for ABE-mediated gene knockout

**DOI:** 10.1016/j.omtn.2026.103014

**Published:** 2026-07-27

**Authors:** James Williamson, Aidin Kazemizadeh, Joanna Jacków-Malinowska

**Affiliations:** 1 St John’s Institute of Dermatology & KHP Centre for Translational Medicine, King’s College London, London, UK

## Main text

**Figure 1:**
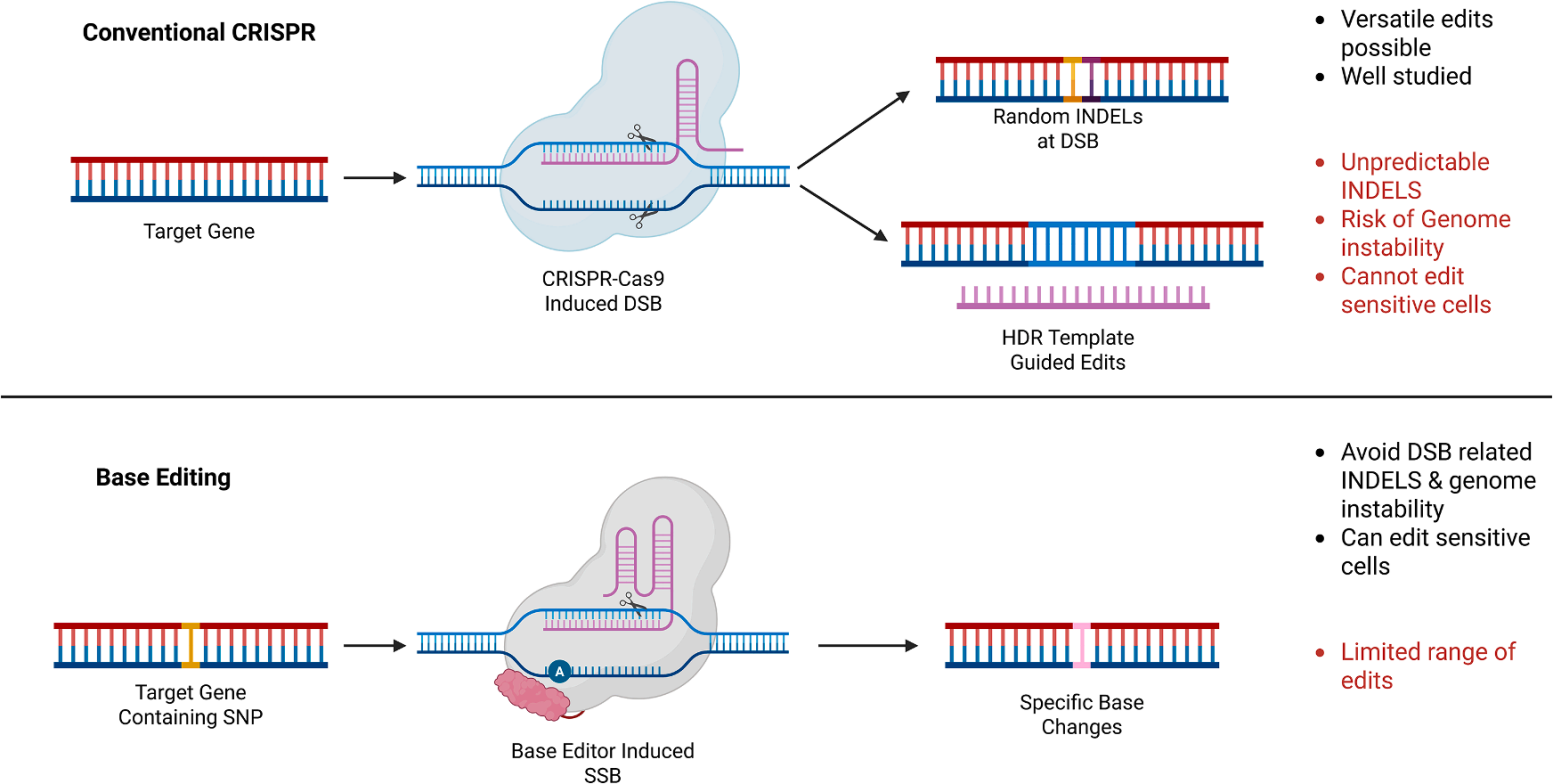
Comparative mechanism and benefits of conventional CRISPR and base editing The editing process for each system is depicted, along with the advantages in black and the disadvantages in red. Made using BioRender.

Since the development of the first base editors, continued evolution has yielded a selection of editors that have been proved to be highly effective and precise in research and their first clinical use.^1^ Built on CRISPR technology, base editors utilize modified Cas proteins fused to a deaminase domain and directed by a single-guide RNA (sgRNA) to a specific genomic locus. Within a defined editing window, the deaminase catalyzes the conversion of one nucleotide to another without generating double-strand breaks (DSBs) (Figure 1). By avoiding DSBs, base editors reduce the risk of random insertions and deletions (indels) and chromosomal rearrangements while minimizing DNA damage responses that can limit editing in sensitive cell types.^2^^,^^3^^,^^4^ Despite their considerable advantages, base editors have not yet been widely adopted, with many laboratories continuing to rely on CRISPR-Cas9 approaches. Several factors may contribute to this trend. In the previous issue of Molecular Therapy Nucleic Acids, Eaton et al. (2026) proposed that a key barrier is the lack of user-friendly computational tools to support experimental design and analysis.^5^ To address this challenge, the authors present a workflow for adenine base editor (ABE)-mediated gene knockout experiments, combining existing design tools with a newly developed analysis platform, MultiEditRbatch.

Focusing on immune-related targets, the pipeline guides users through editor selection, sgRNA design, reagent delivery, and validation of editing outcomes. Because the editing efficiency depends on both the desired nucleotide conversion and local sequence context, selecting an appropriate editor is critical.^6^^,^^7^ The authors highlight BE-Keeper as a useful tool for selecting the most appropriate base editor, recommend BE-Hive for sgRNA design, and suggest SpliceR for targeting splice sites to disrupt gene expression. Given the variability in sgRNA performance, they recommend screening multiple candidate guides in a pilot experiment before progressing to primary cells.

To demonstrate this approach, candidate sgRNAs were initially evaluated in the K562 cell line before being tested in primary human immune cells. This strategy enables the elimination of poorly performing guides, while conserving valuable primary-cell resources. However, the editing efficiency in immortalized cell lines does not necessarily predict performance in primary cells, where chromatin accessibility, cell-cycle dynamics, and intracellular environments differ substantially.^8^^,^^9^ Consistent with this, editing efficiencies varied between K562 and primary cells across several targets, highlighting the importance of validating editing outcomes in the intended cellular context, at a genetic and functional level, particularly when clinical translation is the ultimate goal.

The pipeline was validated across 14 immunologically relevant targets in primary human T and NK cells, achieving high levels of genomic editing and confirmed protein or mRNA depletion for most targets. The diversity of genes examined demonstrates the broad applicability of the approach across different targets and immune cell types. Notably, efficient genomic editing did not always result in functional gene knockout as illustrated by the perforin example, where editing of the exon 1 splice donor failed to abolish protein expression because the exon 1-encoded region is removed during post-translational processing of the mature peptide. This finding highlights the importance of integrating biological knowledge of the target gene with computational design tools when developing base-editing strategies. To facilitate data analysis, the authors developed MultiEditRbatch, a tool that quantifies base-editing outcomes from Sanger sequencing data. By avoiding reliance on next-generation sequencing, the platform provides an accessible and cost-effective solution for laboratories with limited resources. MultieditRbatch is available as both a web application and R package, enabling users with varying levels of computational expertise to analyze multiple samples simultaneously.

Accessibility will become increasingly important as base editors continue to expand in number and specificity. To fully exploit these advances across diverse research fields, computational tools for selecting editing reagents and analyzing editing outcomes must evolve in parallel. Eaton et al. highlight the potential of machine learning-based web tools to predict base-editing outcomes; however, many current models have been trained using a single cell line and delivery modality. As a result, the reproducibility of these predictions in primary cells and *in vivo* settings remains limited, restricting their utility for translational research.

Overall, this article highlights a key challenge facing the field: although base editors have matured into powerful and effective gene-editing tools, their broader adoption remains dependent on the availability of user-friendly design and analysis platforms. By combining practical guidance with newly developed computational resources, Eaton et al. provide an important step toward making base editing more accessible to researchers without extensive gene-editing expertise.

## Acknowledgments

We are grateful for the support of CureEB, DEBRA UK, EB Research Partnership & EB Medical Research Foundations (“EB Charities”), British Skin Foundation, Rosetree Foundation, and EI Cure Project to J.J.-M.

## Author contributions

J.W., A.K., and J.J.-M. contributed equally to conceptualization of ideas and writing.

## Declaration of interests

The authors declare no competing interests.
